# The Radiosensitizing Potential of CC-115, PIK-75 and M3814 DNA-PK Inhibitors for Breast Cancer Targeted Radiotherapy

**DOI:** 10.3390/ijms27219782

**Published:** 2026-10-30

**Authors:** Dragoș Andrei Niculae, Radu Marian Șerban, Doina Drăgănescu, Dana Niculae

**Affiliations:** 1 Faculty of Pharmacy, “Carol Davila” University of Medicine and Pharmacy, 37 Dionisie Lupu Street, 020021 Bucharest, Romania; dragos-andrei.niculae@drd.umfcd.ro (D.A.N.); doina.draganescu@umfcd.ro (D.D.); 2 Horia Hulubei National Institute for Physics and Nuclear Engineering, 30 Reactorului Street, 077125 Magurele, Romania

**Keywords:** DNA-PKcs, NHEJ, copper-64, breast cancer, combination therapy, radiotherapy, DNA damage

## Abstract

NHEJ is one of the preferred DNA-damage-repairing mechanisms for human cells, due to its rapid onset and activity throughout the cell cycle. It is, nevertheless, error-prone and highly relevant in numerous oncological malignancies. In recent years, it has gained a lot of attention as a target either for cancer treatment or as pharmacological- and radio-sensitization approaches. This study evaluated the genotoxicity and radiosensitization potential of three DNA-PK inhibitors (PIK-75, M3814, and CC-115) in breast cancer by comparing MDA-MB-231 and MCF-7 breast cancer cell lines, selected based on reported differences in DNA-repair characteristics and relative NHEJ dependence. Cells were preincubated with the DNA-PK inhibitors, using four different concentrations either in monotherapy or in combination with a DNA damage inducer (doxorubicin or [^64^Cu]CuCl_2_). Viability was measured by MTS assays at 24, 48, and 72 h, while the DNA damage was measured by γH2AX and flow cytometry. DNA-PK inhibitor combinations produced greater reductions in MTS viability in MDA-MB-231 than in MCF-7 under experimental conditions when combined with doxorubicin and ^64^Cu. Combination treatments generally reduced viability relative to inhibitor monotherapy, with clearer concentration dependence and stronger effects in MDA-MB-231. In the case of [^64^Cu]CuCl_2_ alone, MDA-MB-231 viability was reduced to 70–75% at 24 h, whereas several inhibitor combinations reduced it further; MCF-7 showed weaker or inconsistent potentiation. The reduction in metabolic viability became more pronounced at 72 h, particularly in MDA-MB-231 cells. Overall, the results highlight the potential of exploiting repair-pathway dependence to improve targeted radiotherapy in breast cancer.

## 1. Introduction

A critical DNA double-strand break (DSB) repair pathway that operates throughout the cell cycle in order to ensure the genomic stability of the cells is non-homologous end joining (NHEJ) [1,2]. This complex detection and repairing mechanism is capable of repairing DSBs without the need of a homologous template, which makes it a rapid-response mechanism when compared to homologous recombination (HR) [3]. Still, the fast response, a critical parameter to increase survivability, can make this mechanism prone to errors [1,4].

The DNA-dependent protein kinase (DNA-PK) complex is essential for the function of NHEJ, involved in the recruitment and activation of DNA repair factors, such as Artemis, XRCC4, and DNA ligase IV [5,6,7].

As such, NHEJ, which certain cancer types have been reported to greatly rely on for fast DSBs repair, became a target for therapeutic approaches [8]. Using different inhibitors to interfere with the activation of the repair mechanism can make cancer cells more vulnerable to chemical and radiological treatments [9]. Studies have indicated that the presence of DNA-PK inhibitors in cancer cells prior to exposure to radiation can enhance the efficiency of the treatment, or allow the usage of a lower dose that can still have the desired effect [10].

Clinical studies aim to establish a combination therapeutic approach in cancer treatment, by determining how to best exploit the different DNA repair capacities among tumor subtypes, significantly increase the radiosensitivity of cancer cells, while avoiding impacting the healthy tissues, and achieve technological transfer from preclinical studies to applied clinical therapies [11,12,13,14].

PIK-75 is a very potent PI3k and DNA-PK inhibitor [15,16], showing good selectivity for p110α isoform, with IC50s of 2 nM for DNA-PK, and 5.8 nM, 1300 nM, 76 nM and 510 nM for p110α, -β, --γ, and -δ, respectively. It acts as a noncompetitive inhibitor with ATP and competitive with PI substrate. Its strong NHEJ inhibition has been shown in multiple studies in nonasthmatic airway smooth muscle (ASM) cells, asthmatic ASM cells, and lung fibroblasts [17], small-cell lung cancer (SCLC) [18], medulloblastoma [19], acute myeloid leukemia (AML) [20], glioblastoma [21], and mantle cell lymphoma [13], and has even been formulated as HDL-mimetic nanoparticles [16]. PIK-75 has been investigated in vivo as well, showing a reduction in cell invasion in ErbB3WT breast tumor models [22]. Nevertheless, its inhibition of multiple pathways, such as PI3K or p110α, means the biological effects cannot be attributed to DNA-PK blockade alone.

M3814 is a specific anti-DNA-PK ligand, with an IC50 of 3 nM, that was shown in-vitro to strongly block DNA-PK catalytic activity and sensitize cancer cell lines to ionizing radiation (IR) and pharmacological DSB-inducing agents, via rendering the NHEJ pathway incapable of resolving complex DSBs, which leads to an increased number of them and, finally, promoting apoptosis [23]. M3814 was administered orally to two xenograft-bearing mice, on a 6-week fractionated radiation schedule, and it potently bolstered the radiotherapy efficiency, even leading to complete tumor regression using non-toxic dose regimens [23].

CC-115 is a potent, multi-pathway inhibitor that strongly blocks DNA-PK (0.013 µM), mTOR (0.021 µM) and PI3K (0.852 µM) [24], rendering the targeted cell incapable of critical DNA-damage repair. Due to this non-specific mechanism, the observed effects of this compound need to be carefully assessed. It was shown to inhibit DNA-damage repair in CLL cell lines and induce a caspase-dependent cell death path. It was shown to revert CD40-induced chemoresistance, while also blocking cell proliferation [25]. The compound was also tested in vivo in CLL patients, where it showed a decrease in lymphadenopathy. It was shown to cross the blood–brain barrier (BBB) in glioblastoma models, and even allowed one patient to achieve complete remission of endometrial carcinoma for over 4 years [26]. When combined with enzalutamide for metastatic castration-resistant prostate cancer, it showed an additional reduction in prostate-specific antigen (PSA) levels [27]. Another important trait is the very good bioavailability of 53, 76 and 100% in mice, rats and dogs, respectively, displaying a good pharmacokinetic profile [24].

In this study, we investigated the effects these DNA-PK inhibitors have on cells incubated following exposure to different DNA-damaging stress factors. A doxorubicin [28] solution was initially used to induce DNA DSB in order to assess the genotoxic effects the inhibitors would have and establish the adequate working concentration for each inhibitor. We then studied the radiosensitization effects of the inhibitors by exposure to the emissions of copper-64 radioisotope [29,30].

The study aimed to compare the effects of pharmacological DNA-PK inhibition in two breast cancer cell models with reported differences in DNA-repair characteristics and relative reliance on NHEJ-mediated DSB repair. We evaluated the responses of PIK-75, M3814 and CC-115 in monotherapy, as well as in combination with doxorubicin (1.5 µM) and [^64^Cu]CuCl_2_ (20 MBq/mL) by MTS metabolic activity, γH2AX flow cytometry and cell-cycle analysis [31].

## 2. Results

**Figure 1:**
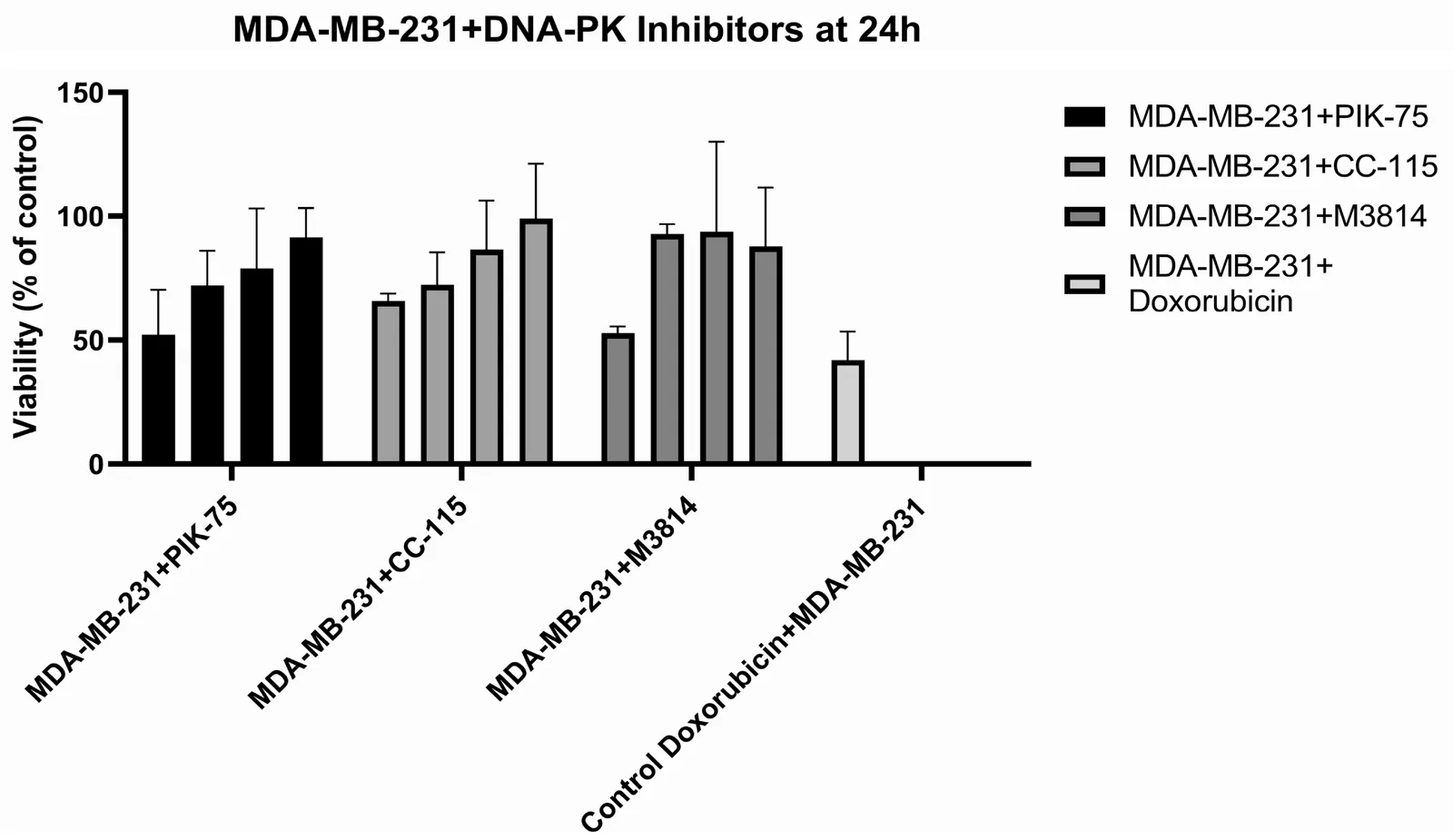

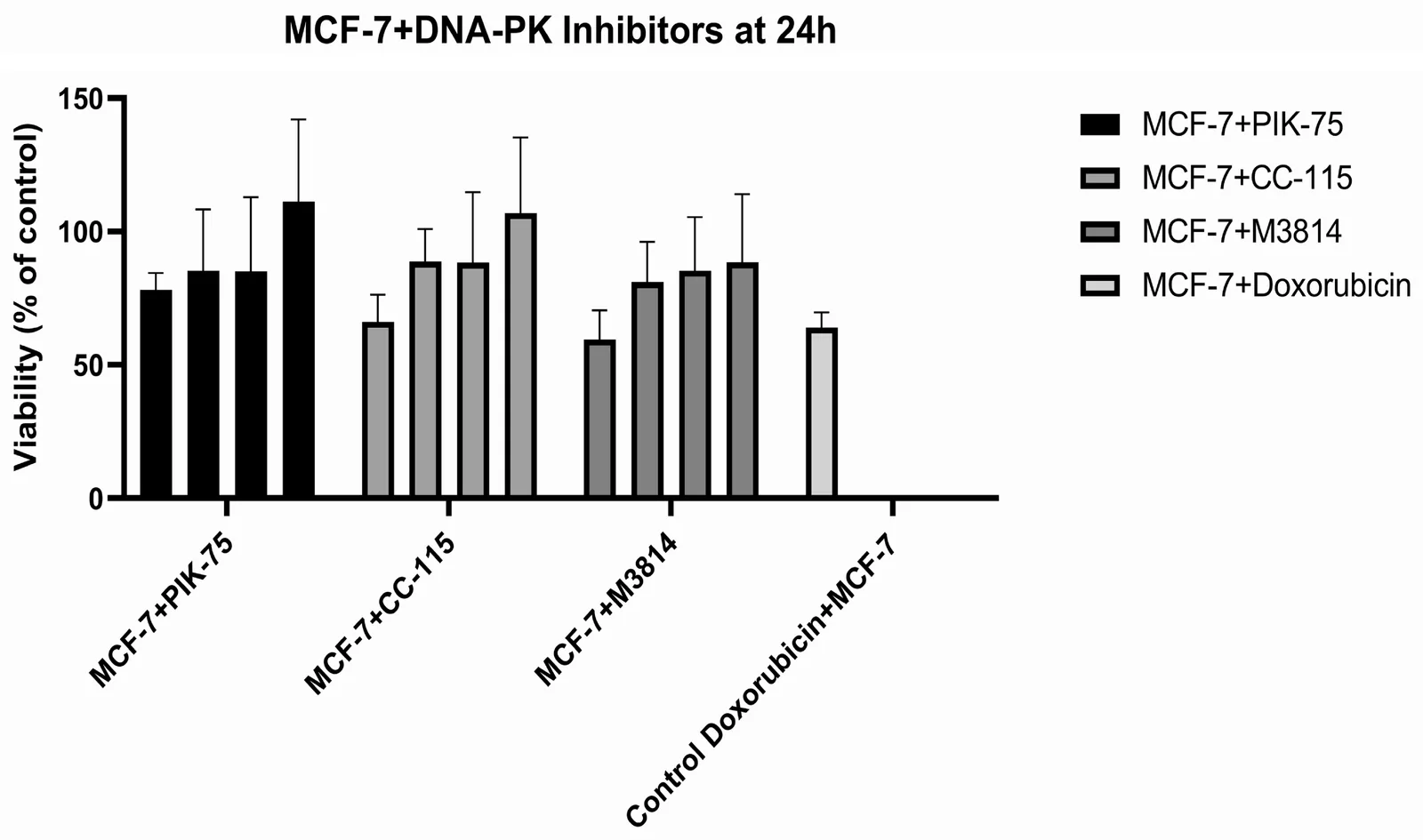
Effect of single therapy of PIK-75 (1 µM, 0.5 µM, 0.1 µM and 0.01 µM), CC-115 (25 µM, 5 µM, 0.5 µM and 0.05 µM) and M3814 (10 µM, 1 µM, 0.1 µM and 0.01 µM) and doxorubicin (1.5 µM) on MDA-MB-231 and MCF-7 normalized to the controls.

For the first experiment, MDA-MB-231 and MCF-7 cell lines were exposed to four different concentrations of PIK-75 (1 µM, 0.5 µM, 0.1 µM and 0.01 µM), CC-115 (25 µM, 5 µM, 0.5 µM and 0.05 µM) and M3814 (10 µM, 1 µM, 0.1 µM and 0.01 µM), the cytotoxic effect being compared to doxorubicin (1.5 µM) (Figure 1). The inhibitors alone had an impact on the viability of MDA-MB-231 cells, with a concentration-related trend for PIK-75 and CC-115. Nevertheless, the impact of the inhibitors is lower than that of doxorubicin, which indicates that the inhibitors used at these concentrations were better tolerated than the already-established pharmacological DSB inducer.

**Figure 2:**
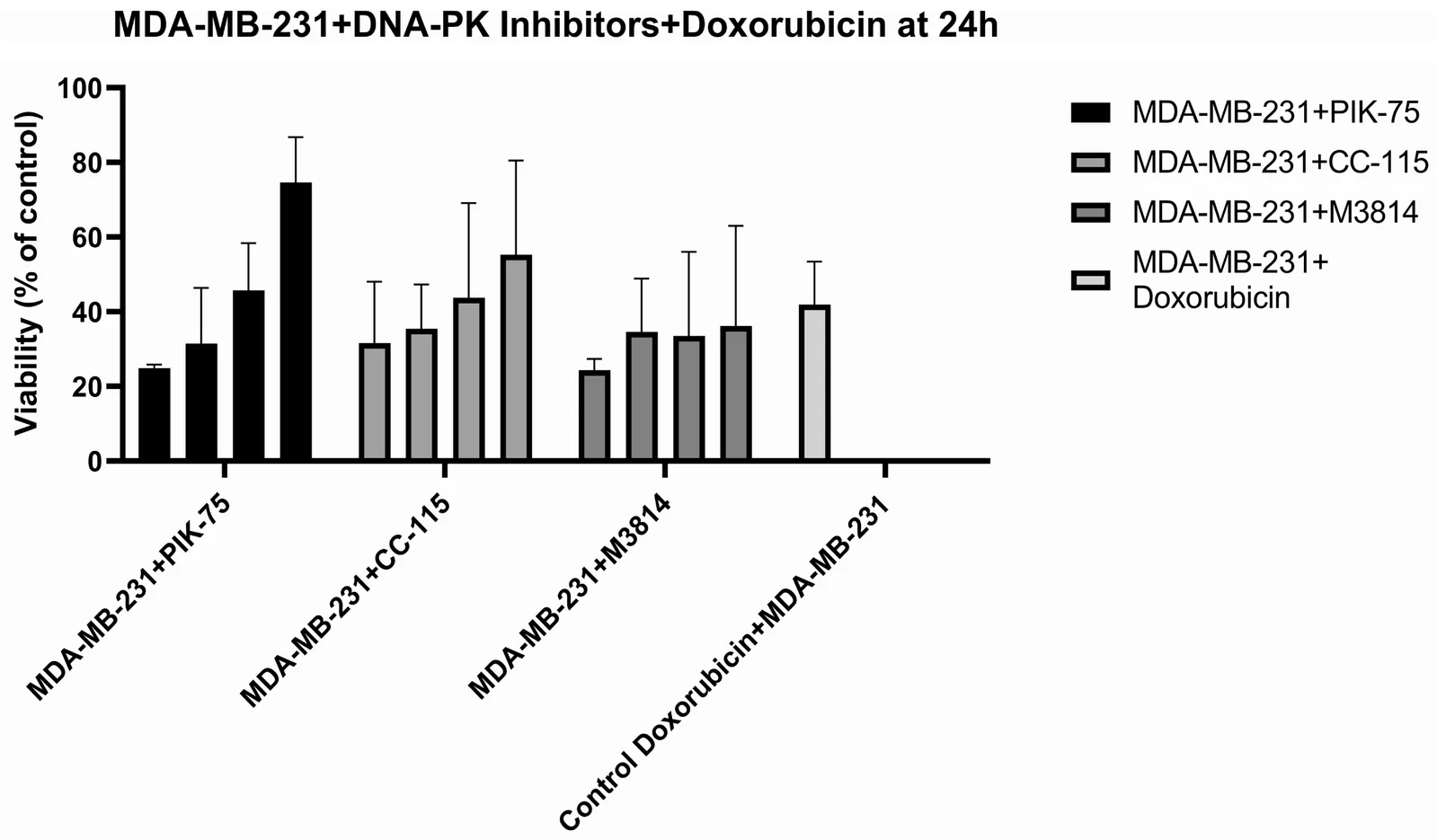

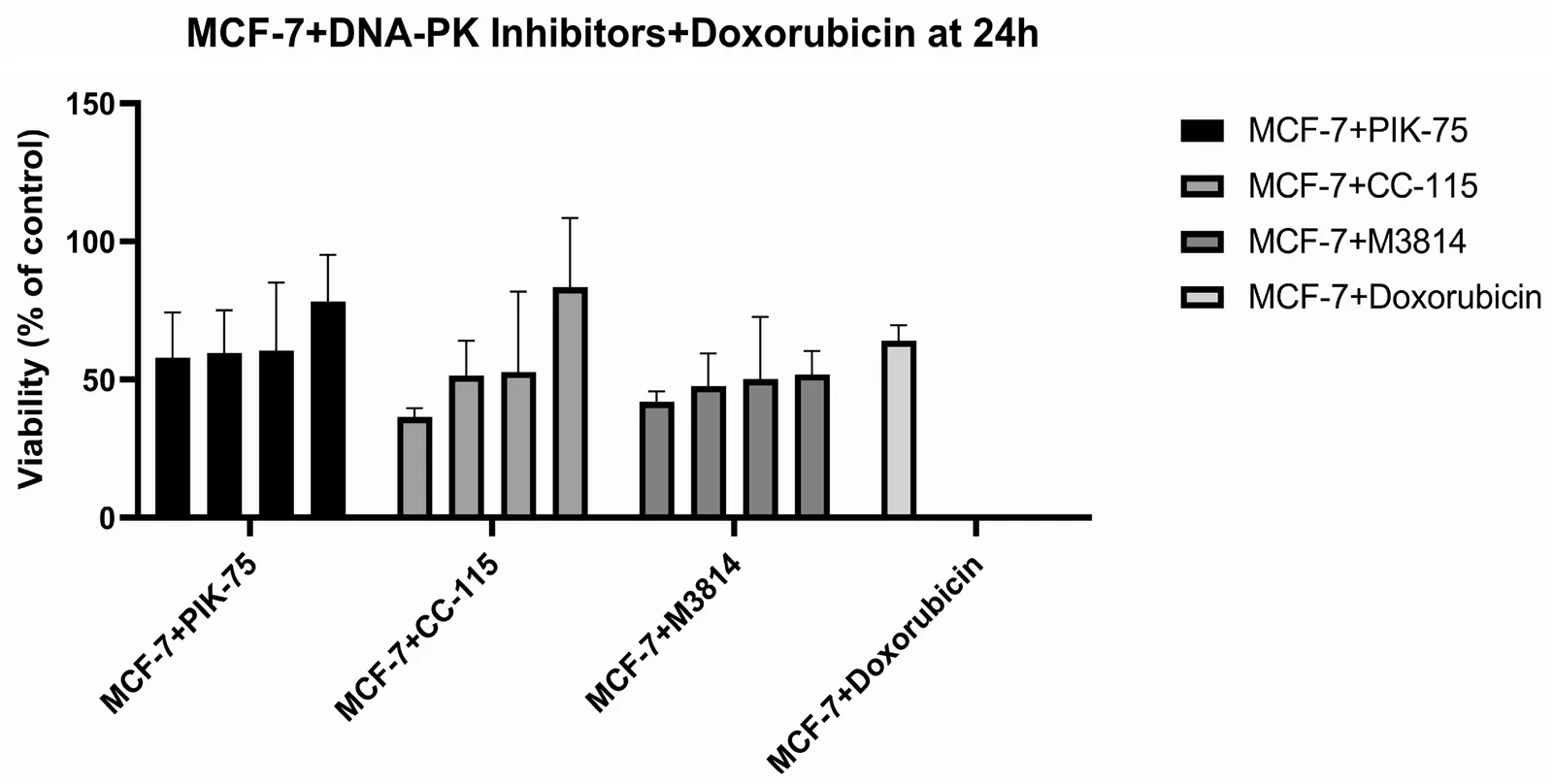
Effect of combination therapy, doxorubicin (1.5 µM) + PIK-75 (1 µM, 0.5 µM, 0.1 µM and 0.01 µM), CC-115 (25 µM, 5 µM, 0.5 µM and 0.05 µM) and M3814 (10 µM, 1 µM, 0.1 µM and 0.01 µM), on MDA-MB-231 and MCF-7, normalized to the controls. The last column shows doxorubicin (1.5 µM) monotherapy.

For the second experiment, the cell lines were exposed to a combination therapy which consisted of the four concentrations of DNA-PK inhibitors with doxorubicin (1.5 µM) (Figure 2). The cell viability further dropped compared to either the inhibitor or doxorubicin alone. For the MCF-7 cell line, we noticed a lower degree of differentiation between the concentrations. Nevertheless, even in this case, we could see an additional effect when doxorubicin was combined with the DNA-PK inhibitors. In the case of the MDA-MB-231 cell line, the impact was clearly higher, while the concentration–effect trend was clearer. PIK-75, the most potent inhibitor selected, showed a concentration-dependent impact on cells’ viability.

**Figure 3:**
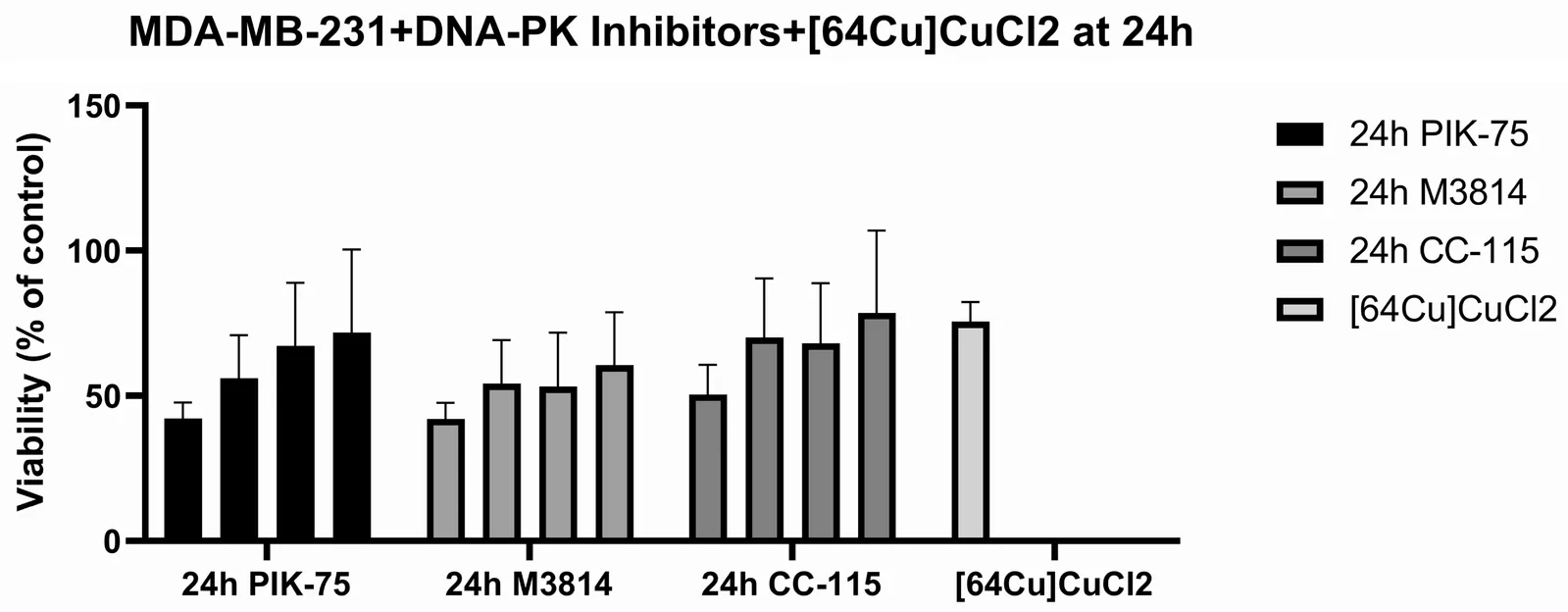

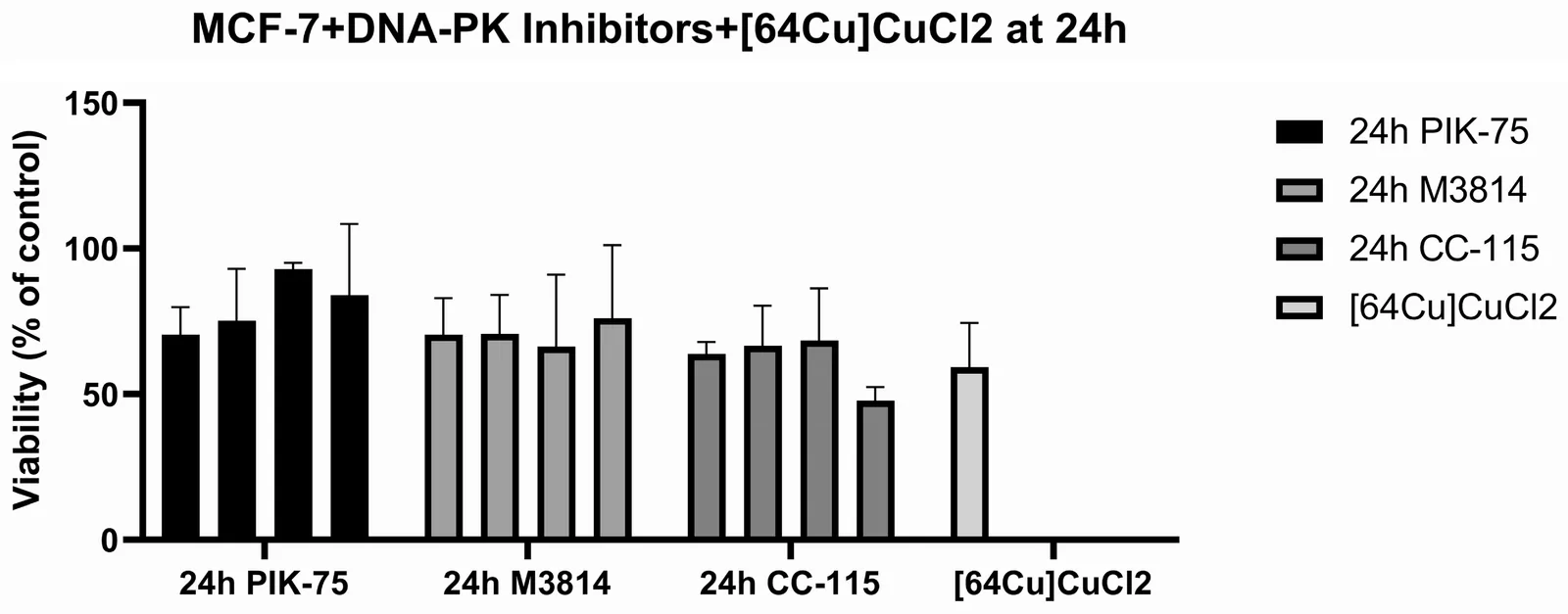
Effect of combination therapy, [^64^Cu]CuCl_2_ (20 MBq/mL) + PIK-75 (1 µM, 0.5 µM, 0.1 µM and 0.01 µM), CC-115 (25 µM, 5 µM, 0.5 µM and 0.05 µM) and M3814 (10 µM, 1 µM, 0.1 µM and 0.01 µM), on MDA-MB-231 and MCF-7, normalized to the controls. The last column shows the effect of [^64^Cu]CuCl_2_ (20 MBq/mL) monotherapy.

For the third part of the experiment, the cell lines were subjected to a combination therapy of [^64^Cu]CuCl_2_ and DNA-PK inhibitors, using the four different concentrations of inhibitors, the same as before (Figure 3). A more consistent concentration-dependent impact was observed for MDA-MB-231 when exposed to PIK-75, as in the case of the other two DNA-PK inhibitors, differences appear between concentrations, especially for C1 and C4, while the C2 and C3 show similar outcomes. Some inhibitor-treated MCF-7 conditions produced MTS signals comparable to or higher than [^64^Cu]CuCl_2_ monotherapy. For MCF-7, the response pattern was flatter, with no clear correlation between concentration and effect.

**Figure 4:**
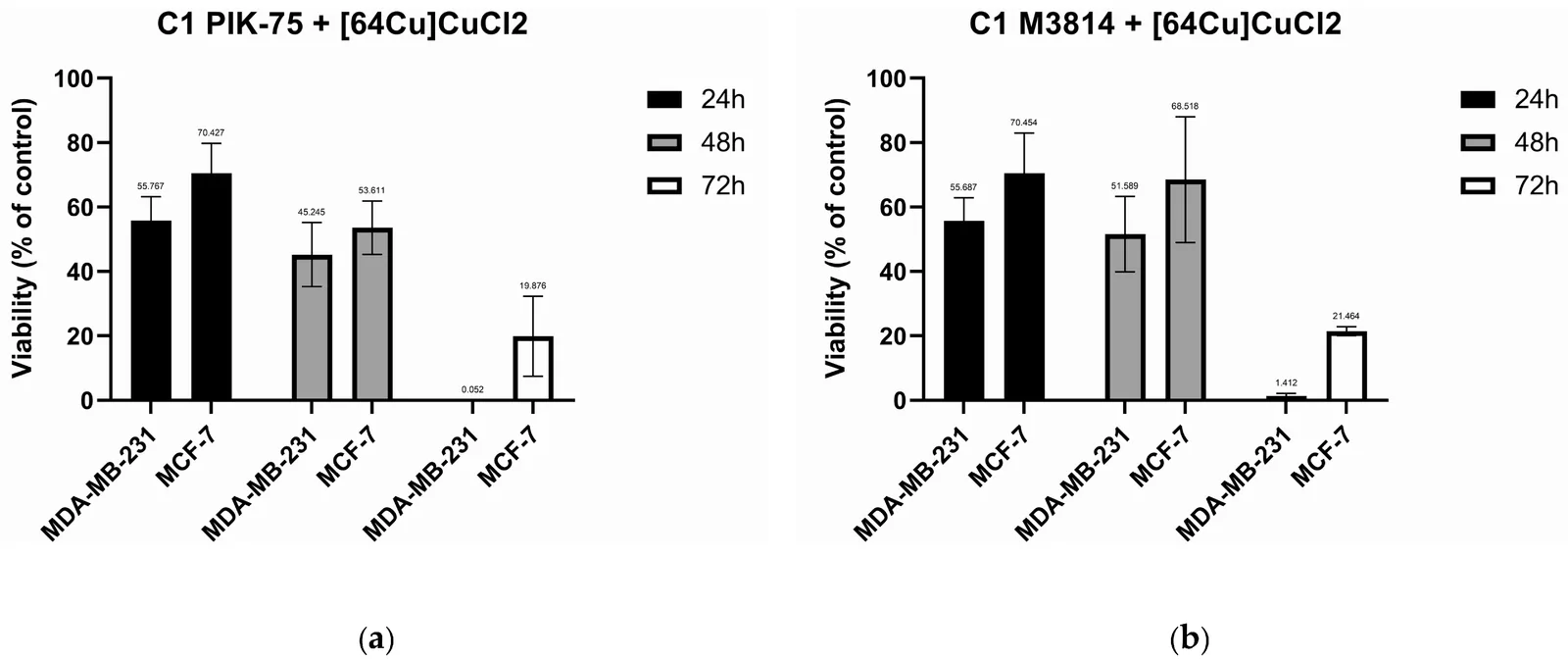

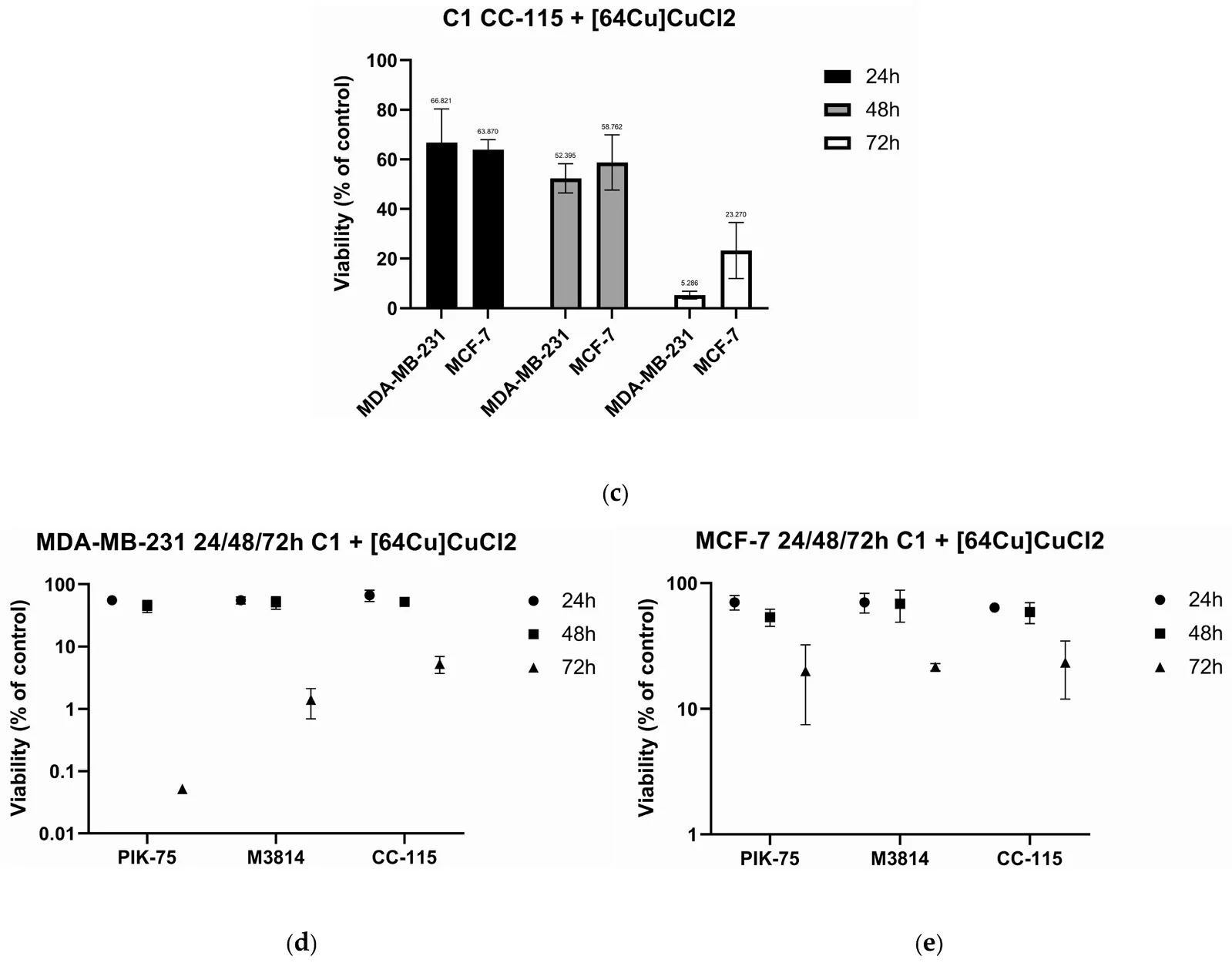
(**a**) Comparison of the PIK-75 + [^64^Cu]CuCl_2_ (20 MBq/mL) effect between cell lines. (**b**) Comparison of the M3814 + [^64^Cu]CuCl_2_ (20 MBq/mL) effect between cell lines. (**c**) Comparison of the CC-115 + [^64^Cu]CuCl_2_ (20 MBq/mL) effect between cell lines. (**d**) Logarithmic scale showing the dose–effect curve of the DNA-PK inhibitors + [^64^Cu]CuCl_2_ (20 MBq/mL) in the MDA-MB-231 cell line. (**e**) Logarithmic scale showing the dose–effect curve of the DNA-PK inhibitors + [^64^Cu]CuCl_2_ (20 MBq/mL) in the MCF-7 cell line. The logarithmic scale was used to show, where possible, the error bars that are not visible in the linear scale.

All experiments included three time-points, 24, 48 and 72 h, in order to better understand the manner in which the cells adapt to the inhibitor’s blockade. DNA-PK inhibitors’ C1 was selected for representation over all time-points due to having the best radiosensitization effect (Figure 4).

**Figure 5:**
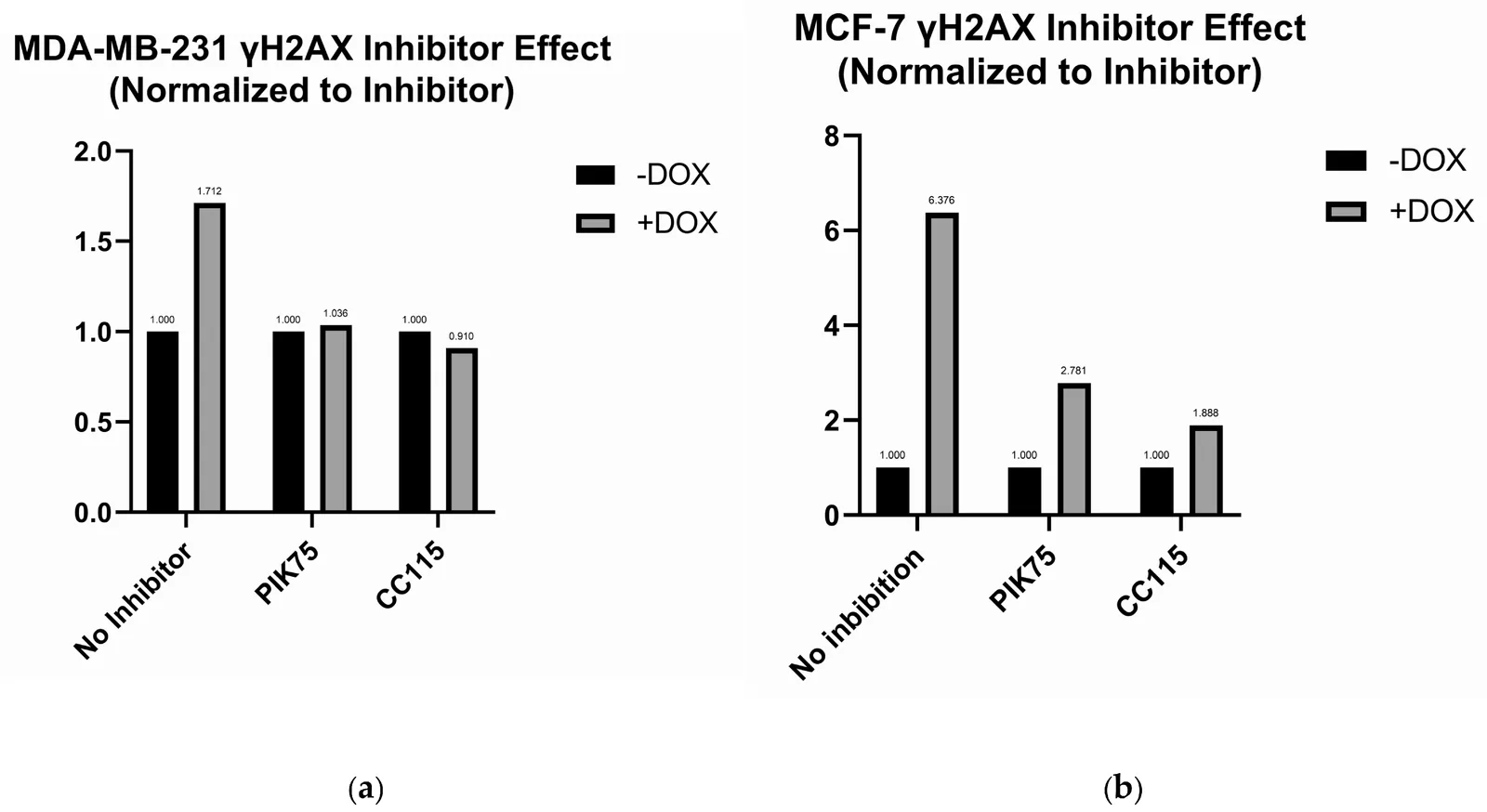
γH2AX results at 4h: (**a**) γH2AX analysis of the MDA-MB-231 cell line following incubation with PIK-75 (0.5 µM), CC-115 (5 µM) and doxorubicin (1.5 µM); (**b**) γH2AX analysis of the MCF-7 cell line following incubation with PIK-75 (0.5 µM), CC-115 (5 µM) and doxorubicin (1.5 µM). Results here are reported according to their respective DNA-PK inhibitors.

We used the γH2AX assay to assess the level of DNA damage induced in the breast cancer cell lines post-incubation with the DNA-PK inhibitors, either alone or in combination with doxorubicin. M3814 was also originally intended for inclusion in the γH2AX experiment; however, technical problems affecting the available stock solution prevented reliable use of the compound in this experimental series. We analyzed γH2AX signal levels following 4 h of incubation of the two cell lines with CC-115 5 µM and PIK-75 0.5 µM, both with and without the presence of doxorubicin. The results are presented in Figure 5.

For MCF-7, in the presence of doxorubicin, the γH2AX signal increases; the clearest increase was observed for PIK-75 in combination with doxorubicin. For the MDA-MB-231 cell lines, both in the presence of doxorubicin and in the control, we noticed an increase in γH2AX phosphorylation, similar to the other cell line. The presence of DNA-PK inhibitors strongly affected the γH2AX assay for MDA-MB-231. When incubated with PIK-75 inhibitor, the level of γH2AX phosphorylation was slightly increased by the presence of doxorubicin, while the combination of doxorubicin and CC-115 caused a slight decrease in the γH2AX phosphorylation.

**Figure 6:**
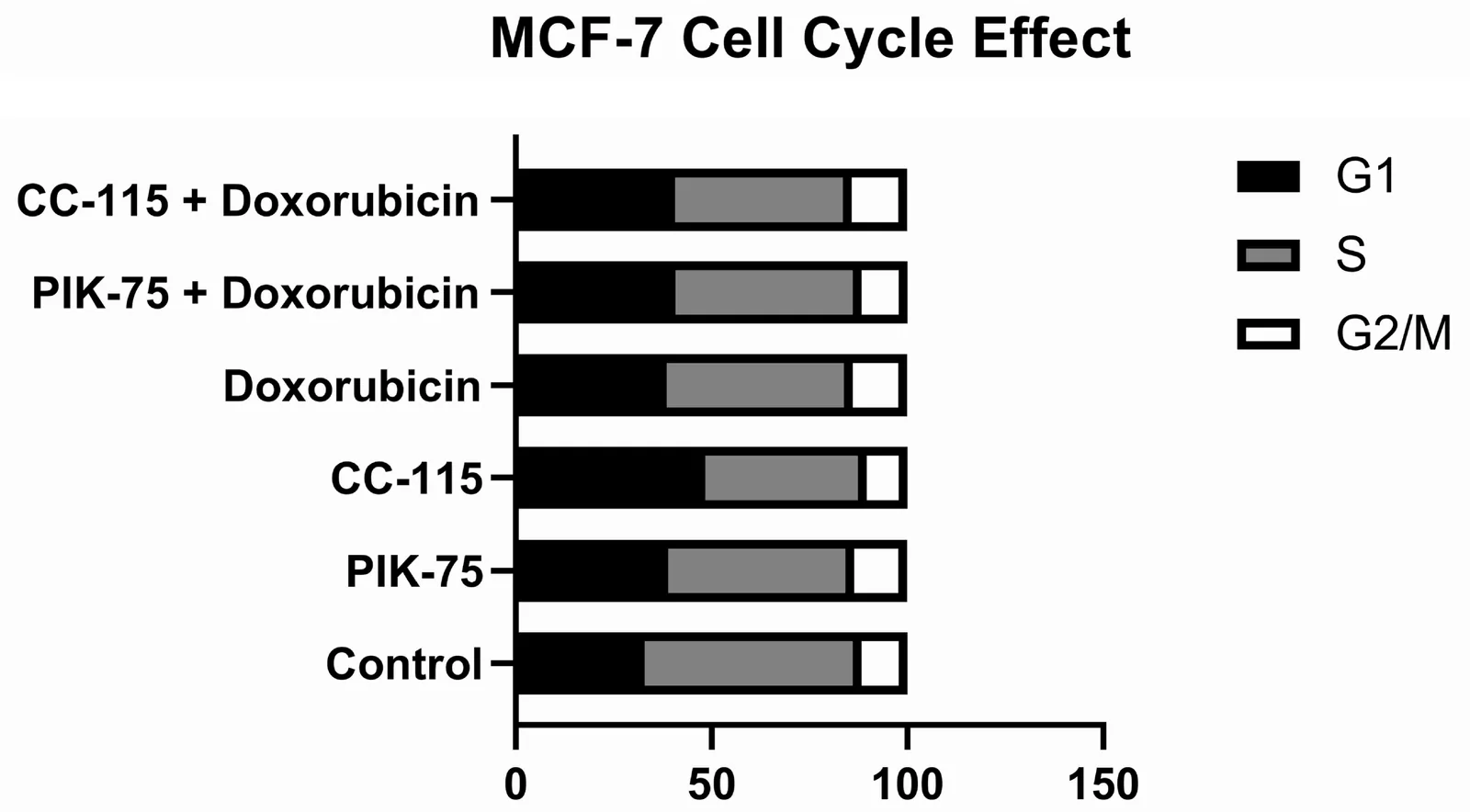
Cell-cycle distribution for MCF-7 at 4h. The results are normalized to the total number of detected cells.

In MCF-7, the cell-cycle profile at 4h appears relatively stable across treatments (Figure 6). The control has a large S-phase fraction (48%) and a small G2/M fraction (10.2%), and neither DNA-PK inhibitors nor doxorubicin seem to produce a dramatic shift toward G2/M. This trend is available for the inhibitor–doxorubicin combinations as well. This suggests that, at the analyzed time-point, MCF-7 does not undergo a strong checkpoint-mediated cell-cycle arrest after these treatments. That fits reasonably well with the viability data, where MCF-7 showed a more resistant and less dose-responsive phenotype. If γH2AX positivity is present but not accompanied by a major G2/M accumulation, it could indicate that MCF-7 tolerates or is still able to repair the damage without being forced into arrest, possibly through the HR-mediated repair capacity.

**Figure 7:**
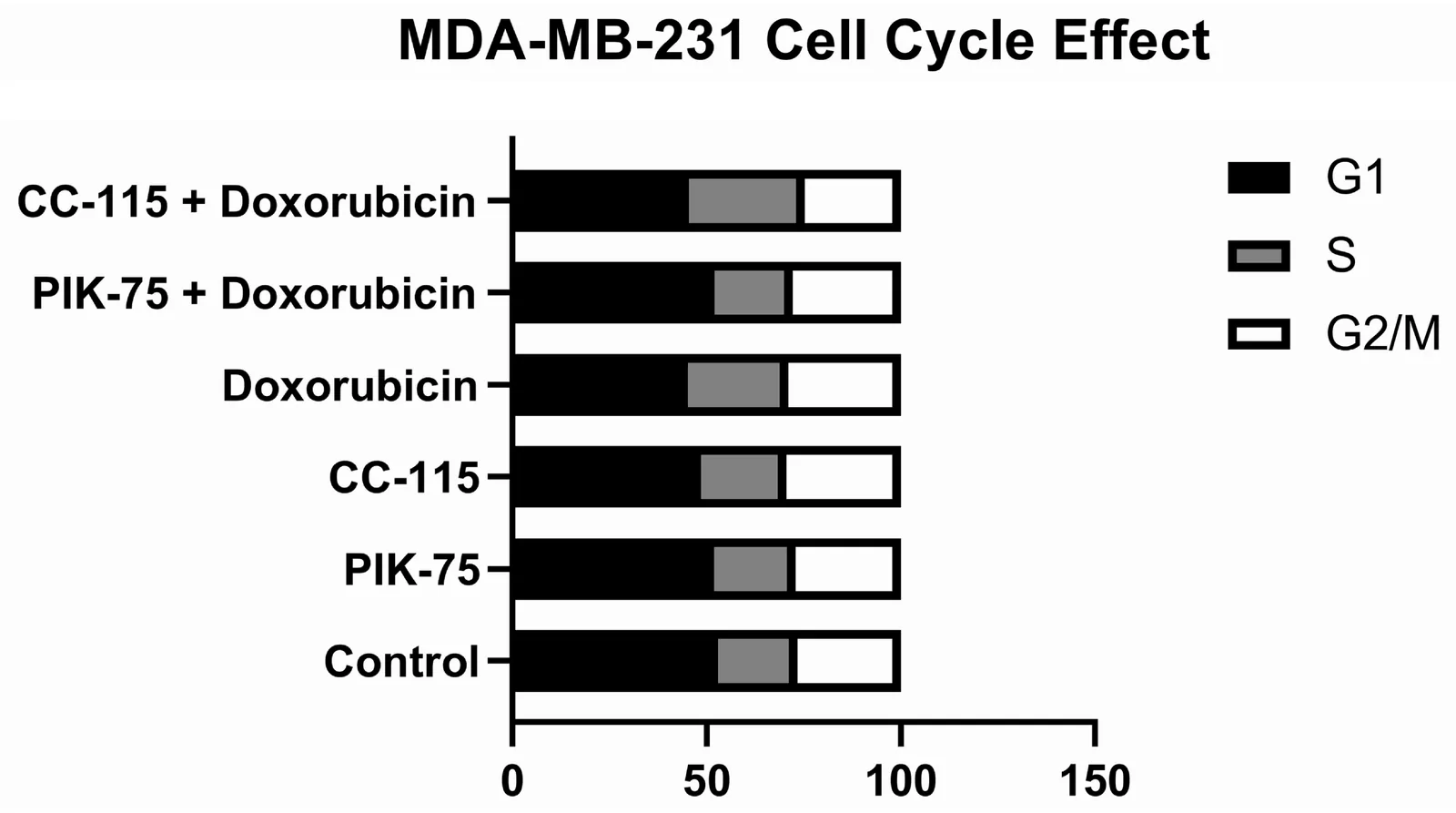
Cell-cycle distribution for MDA-MB-231 at 4h. The results are normalized to the total number of detected cells.

In MDA-MB-231, the cell cycle at 4h control distribution (Figure 7) already shows a larger G2/M (27%) compartment than MCF-7 (10.2%), a trend that is consistent across the treated groups. However, the treatment-induced changes are modest; doxorubicin alone and in combination with the inhibitors does not seem to produce clear additional G2/M accumulation compared to the control. This is interesting due to MDA-MB-231 viability data showing a much more strongly delayed cytotoxicity, especially at 72 h with [^64^Cu]CuCl_2_ combinations. Based on the delayed reduction in MTS viability that is not accompanied by a pronounced early redistribution of the cell-cycle profile, the loss of viability in MDA-MB-231 may not be explained simply by an early cell-cycle arrest, but rather by the accumulation of unrepaired or misrepaired DNA lesions that later could be translated into apoptotic death, or loss of proliferative capacity.

**Figure 8:**
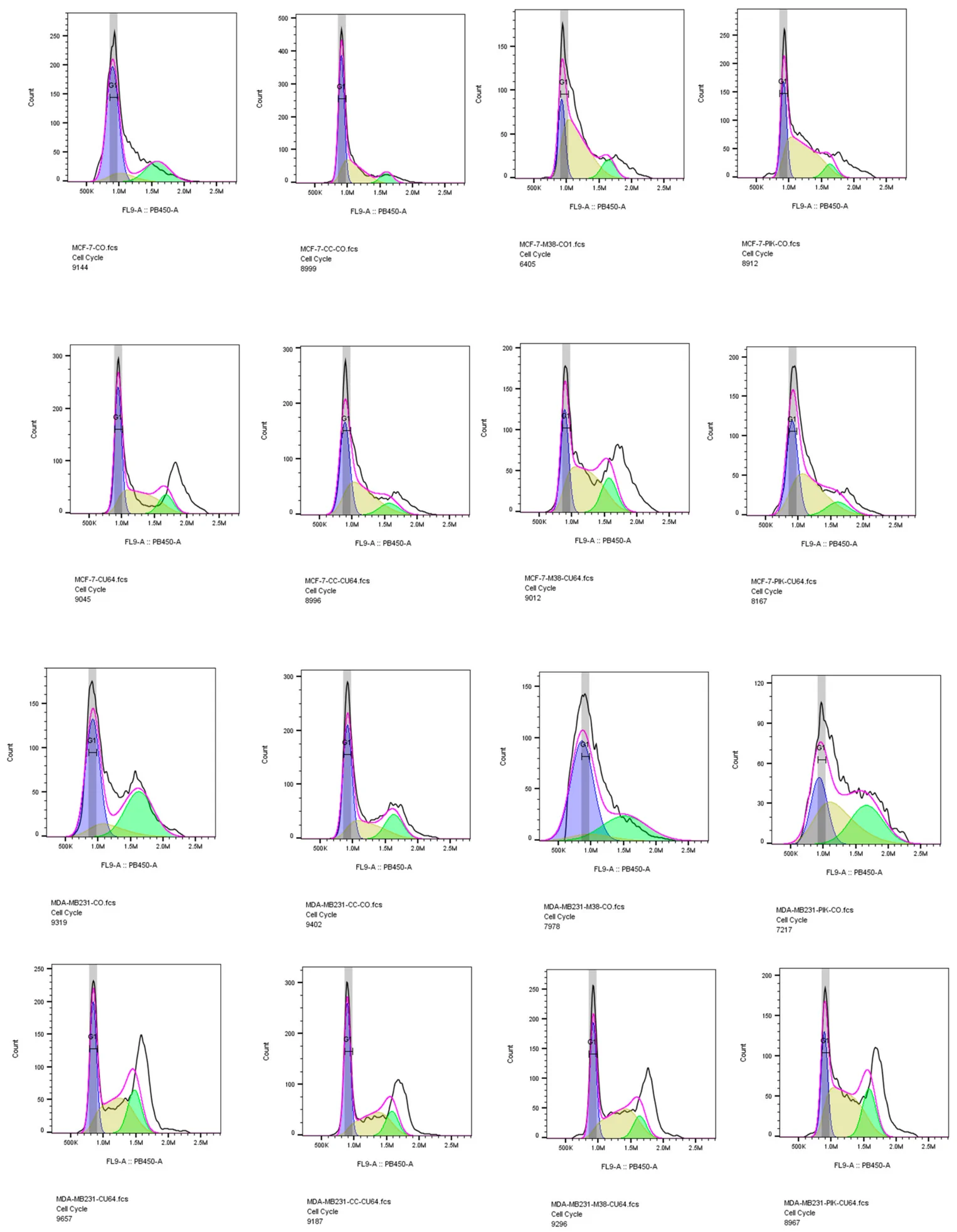
γH2AX flow cytometry histograms.

The original γH2AX flow cytometry histograms are presented in Figure 8, while the percentages corresponding to the G0/G1, S, and G2/M populations are presented in Table 1.

## 3. Discussion

Different response patterns were observed between the two cell lines across the 24h viability data. These differences are consistent with previously reported differences in their DNA-repair characteristics, specifically the HR reliance. MDA-MB-231 shows a more coherent concentration-related response to DNA-PK inhibition, especially when the inhibitors are combined with DNA-damaging treatments, whereas MCF-7 displays a flatter and more heterogeneous profile, with broader overlap between error bars and less consistent dose-dependent inhibition. A broader precedent for modifying treatment sensitivity through interference with DNA-damage and stress-response pathways has also been reported in breast cancer models [32]. This is biologically plausible if MDA-MB-231 is treated as the more NHEJ-reliant model; thus, blocking DNA-PK should compromise the key DSB-repair route. Published data also support that MDA-MB-231 can show low HR capacity and sensitivity to DNA-PK inhibition in the context of repair defects [33], while this study did not assess RAD51 recruitment, 53BP1, BRCA-associated repair efficiency or DNA-PK phosphorylation. DNA-PK is central to NHEJ-mediated DSB repair, and its inhibition is expected to increase vulnerability to DSB-inducing agents.

The [^64^Cu]CuCl_2_ combination therapy suggests a greater combination-associated effect in MDA-MB-231 than in MCF-7. In MDA-MB-231, [^64^Cu]CuCl_2_ alone maintains viability around the ~70–75% range, while several inhibitor + [^64^Cu]CuCl_2_ conditions reduce viability further, indicating that DNA-PK inhibition may impair the repair of radiation-induced DNA damage. This is consistent with the therapeutic rationale of [^64^Cu]CuCl_2_, which combines β^−^ and Auger electron emissions and has been shown to induce changes in viability, DNA damage, oxidative stress, and stress-response pathways in cancer cells [30]. In contrast, for MCF-7, the inhibitor + [^64^Cu]CuCl_2_ combinations do not consistently reduce viability below [^64^Cu]CuCl_2_ in monotherapy; in several conditions, viability is actually higher than with radiocopper monotherapy. One possible explanation is that, at 24 h, MCF-7 may compensate more efficiently through HR or other DDR mechanisms, making Cu-64 radiosensitization by DNA-PK inhibition weaker or more inhibitor-specific; however, pathway utilization was not directly assessed in the present study.

Doxorubicin combinations produced lower MTS viability than doxorubicin alone under several experimental conditions, particularly in MDA-MB-231. The effect is also visible in MCF-7, especially for M3814 and some CC-115 conditions, although the magnitude is less uniform than in MDA-MB-231. Mechanistically, this fits well with the known role of DNA-PK inhibitors as enhancers of radiation- and pharmacological-induced DNA damage; preclinical studies with selective DNA-PK inhibition have shown potentiation of both ionizing radiation and doxorubicin effects [28]. However, from viability data alone, the synergetic effect should ideally be confirmed quantitatively using further analyses.

Regarding the inhibitors, M3814, being also the most selective DNA-PK inhibitor from the studied selection, appears to imprint one of the strongest inhibitory effects in its higher concentrations; thus, there is a big yet unelucidated plateau-like structure for the immediate smaller concentrations, with the broad concentrations’ spacing imprinting limits to the interpretation. In the future, we should investigate intermediate concentrations for the most promising candidate, to get a clearer image of the dose–effect curve. CC-115 may produce a broader effect due to its cumulative blockade of both DNA-PK and mTOR, meaning its cytotoxicity may reflect simultaneous impairment of DSB repair and survival/growth signaling. This may explain why it performs well in some doxorubicin combinations but less uniformly with [^64^Cu]CuCl_2_. PIK-75, as a less selective DNA-PK-targeting compound, that also inhibits the PI3K and p110α activity, is harder to interpret because part of its effect may come from PI3K pathway inhibition rather than DNA-PK blockade alone; therefore its effects cannot be attributed solely to NHEJ inhibition. Overall, the data support the hypothesis that DNA-PK inhibition preferentially sensitizes the reported NHEJ-reliant MDA-MB-231 model, especially to doxorubicin and radiation-induced damage, while MCF-7 shows a more resistant phenotype, possibly due to retained HR capacity and compensatory DDR activity.

Time-course analysis revealed that the cytotoxic interaction between DNA-PK inhibition and [^64^Cu]CuCl_2_ is delayed and becomes markedly significant at 72 h, particularly in the MDA-MB-231 cell line. While both MCF-7 and MDA-MB-231 maintained relatively high viability at 24–48 h, MDA-MB-231 underwent a profound loss of viability by 72 h, supporting the hypothesis that DNA-PK inhibition compromises the repair of radiation-induced DNA damage in the reported NHEJ reliant model. In contrast, the more HR-competent MCF-7 model showed only partial delayed sensitivity, suggesting compensatory DNA repair capacity or a higher threshold for radiation-induced lethality.

The marked reduction in viability observed at 72 h is consistent with the delayed biological manifestation of unresolved DNA damage, and a difference must be made between this and the fast kinetics of DSB repair. Since canonically the innate DSB repair mechanisms generally occur within 1–2 h, for NHEJ-mediated repair, or more than 8 h, for the HR-mediated mechanism [34], persistent damage beyond 24 h is commonly interpreted as residual or complex lesions that are difficult to repair. It was shown that retention of γH2AX signal 24 h after DNA-damaging treatment closely correlated with the loss of clonogenic survival, and cells retaining RAD51 foci 12–24 h after irradiation were likely to die afterwards [35,36]. Multiple studies highlight that apoptotic DNA fragmentation becomes observable from 48 h onward, with clear apoptotic biological effects at 72 and even 96 h [37,38]. Additionally, DNA-PK blockade was shown to prolong this window, where the return to baseline for radiation-induced γH2AX/53BP1/pATM foci is around 6 h, but when DNA-PK inhibition is also used in combination with radiation-induced DNA damage, these foci persisted at least until 72 h, together with micronuclei and prolonged G2/M arrest [28]. The significant 72 h viability collapse in MDA-MB-231 therefore supports the hypothesis that DNA-PK inhibition compromises the repair of [^64^Cu]CuCl_2_-induced DNA damage, leading in the end to cell death.

The results of the γH2AX assay, a dynamic DNA-damage signaling marker, on the MDA-MB-231 cell line could be caused by the potential interaction between the DNA-PK inhibitors and the phosphorylation kinetics of γH2AX foci, but without definitive proof, this remains a hypothesis. The method detects rapid phosphorylation in the histone H2AX’s surrounding molecules at serine-139 by PI3K-like kinases as a response to DSBs, but it is not a direct quantitative measure of unrepaired DSBs. The interference of the DNA-PK inhibitors with the activity of those kinases could be more prominent in the case of NHEJ-dependent cells like MDA-MB-231, compared to MCF-7.

Previous studies have indicated that the presence of DNA-PK inhibitors caused a decrease in γH2AX signal following γ irradiation of RIC1 cells, with no other significant effects on the cells [39,40]. We noticed a similar interaction in the case of the MDA-MB-231 cells incubated with CC-115 inhibitor, with and without doxorubicin, with the inhibitor’s presence causing a slight decrease in the γH2AX following exposure to the DNA-damaging compound. Additionally, a 4h time-point measurement is insufficient to resolve the kinetics of γH2AX phosphorylation. Thus, future orthogonal experiments involving the most promising candidate will focus on overcoming the barrier DNA-PK inhibitors pose for the γH2AX assay, extend the measured timepoints to include earlier as well as later measurements and analyze DNA damage by other methods, such as comet assay, 53BP1 and RAD51 where relevant [41,42].

These preliminary findings show a clear difference between the effects DNA-PK inhibition had on two breast carcinoma cell lines, based on their reported difference in NHEJ dependency, and further offers a rational path to enhance radiotherapeutic efficacy by exploiting tumor’s genotypic vulnerabilities.

Considering current results, further studies will focus on multiple intermediate concentrations of M3814 in order to assess its radiosensitizing potential by clonogenic survival tests, orthogonal DNA-damage assays, and apoptosis characterization where appropriate, creating the framework for future in vivo characterization, looking for a better description of the impact on cellular sensitivity to genotoxic stress induced by DNA-damaging compounds and already-established radiotherapeutic treatment options, especially higher-energy β^-^ emitters and higher LET radiation.

There are current limitations for this study. Only cell viability was measured through the MTS reduction test, while clonogenic survival, which still remains a gold standard, was not included in this study stage and will be employed for the in-depth characterization of the most promising candidate, M3814. The concentration intervals were relatively broad; future studies concentrating on just one inhibitor will include more concentrations tested so the dose-curve will be clearer. Formal synergy analysis using Bliss/Loewe or similar methods was not performed and is key to completely comprehending the synergy potential of the combinations. The study was exploratory, as it is intended to hierarchize these three DNA-PK inhibitors in two breast cancer cell lines that had different reported DNA-repair mechanisms profiles in order to set the foundation for a more comprehensive analysis of the candidate that shows the most translational potential, and was not designed for extensive inferential comparisons. The HR and NHEJ pathway activation or DNA-PK engagement was not measured directly. γH2AX was evaluated only for PIK-75 and CC-115 and using just one time-point. As the timing of γH2AX, and the cell-cycle analysis, appears to be highly cell-line-dependent, multiple time-points will be included in the next stage, as well as orthogonal DNA-damage assays. No in vivo validation was performed, as this critical aspect was outside the scope of this initial in vitro study.

## 4. Materials and Methods

### 4.1. DNA-PK Inhibitors

The inhibitors used in this study are PIK-75, M3814 and CC-115, obtained from Selleckchem (Cat. No. S1205, S8586, and S7891, respectively). Stock solutions were prepared using dimethyl sulfoxide (DMSO); final concentrations used in the study were obtained through dilution with phosphate buffer solution (PBS). The chosen concentrations were based on reported inhibitory potency and IC50 values, previously published in vitro concentration ranges as well as the need for an exploratory concentration range spanning multiple orders of magnitude. The selected concentrations were intended for comparative in vitro screening and should not be interpreted as clinically achievable systemic exposures.

### 4.2. Cell Cultures

Two cell lines were used, MDA-MB-231 and MCF-7. MDA-MB-231 cells were cultivated in RPMI culture media supplemented with 10% fetal bovine serum (FBS) and 1% antibiotic solution, while MCF-7 cells were cultivated in DMEM culture media with 10% fetal bovine serum (FBS), 1% antibiotic solution and non-essential amino acids.

Cell samples were prepared 24h before experiments to ensure adherence.

### 4.3. Incubation Experiments

Cells samples were pre-incubated with 4 different concentrations of DNA-PK inhibitors (Table 2) one hour before the DNA damaging stress factor was introduced, either doxorubicin 1.5 µM or [^64^Cu]CuCl_2_ (20 MBq/mL), to ensure their internalization in the cells. The cells were incubated for 24, 48 and 72 h, with the DNA-PK inhibitor and the DNA-damaging agent present. [^64^Cu]CuCl_2_ solution was obtained and purified as in previous experiments [30]. The maximum volume used for DNA-PK inhibitor solution was up to 10% of well culture media volume.

### 4.4. Viability

Cell viability was investigated using an MTS assay kit, CellTiter 96^®^ AQueous One Solution Cell Proliferation Assay kit (Promega Corporation, Madison, WI, USA), using the protocol recommended by the manufacturer, in triplicate. The DNA-PK inhibitors’ effect on cell viability was tested at 24h, 48 h and 72 h following exposure to DNA-damaging factors. At each experimental time, MTS reactive was added to the culture media, incubated for 1h, followed by reading the optic density (OD) at 490 nm. The results were expressed as percentage of control cell (untreated cells) viability ± standard error of the mean.

### 4.5. γ. H2AX

Genotoxicity was investigated using the γH2AX method at 4h. Following DNA-PK inhibitor incubation (PIK-75 0.5 µM; CC-115 5 µM) and exposure to DNA-damaging factors (doxorubicin 1.5 µM), samples were detached, centrifuged at 2000 rpm for 5 min, and resuspended in 1 mL cold ethanol. Celltrace Oregon Green 488 (Invitrogen Thermofisher refC34555, Waltham, MA, USA) was used for barcoding. The barcoded cell samples (cells treated with doxorubicin) were incubated for 30 min with 1 mL of PBS with Celltrace Oregon Green 488 in the dark, tapping each 5–10 min to keep the cells resuspended. Sample cells were resuspended with 10 mL of PBS with 2% FBS, centrifuged at 2000 rpm for 5 min, then resuspended in 6 mL of PBS with 2% FBS. An equal number of barcoded cell samples was added to each test sample cell tube, followed by centrifugation at 2000 rpm for 5 min, then resuspension in 50 µL of detergent buffer with 4% (*w*/*v*) nonfat milk for 5 min to block nonspecific binding. Subsequently, 50 µL of detergent buffer with 4% (*w*/*v*) nonfat milk containing 0.125 µg of mouse-antihuman H2AX (Ser139) antibody labeled with eFluor 660 (Invitrogen Thermofisher Catalog # 50-9865-42) was added to the cell samples for 1 h incubation at room temperature. Then, 10 mL of PBS with 2% FBS was added to the samples, followed by centrifugation at 2000 rpm for 5 min and resuspension in 200 µL of Hoechst 33258 solution. Samples were analyzed using a CytoFLEX Flow Cytometer (Beckman Coulter Life Sciences, Indianapolis, IN, USA). The results were normalized to untreated cells without the mouse-antihuman H2AX (Ser139) antibody labeling.

## 5. Conclusions

Our preliminary findings outline a difference between the effects of DNA-PK inhibitors on two cell lines of breast carcinoma, in line with the reported difference in cellular dependence on the NHEJ pathway tasked with the response and repair of DSBs. Several DNA-PK inhibitor combinations produced greater decreases in measured cellular viability than the corresponding monotherapies, the effects being more consistent in the MDA-MB-231 cell line. This NHEJ dependency is a potential prime target for developing genotypic-specific therapeutic approaches in combination with chemotherapy and radiotherapy.

Future experiments will focus on multiple intermediate concentrations of M3814 in order to assess its radiosensitizing potential by clonogenic survival tests, orthogonal DNA-damage assays, and cell death characterization, creating the base for future in vivo validation and creating a clearer image of the potential radiotherapeutic adjuvancy these DNA-repair inhibitors can bring to already established radiotherapeutic platforms as higher-energy β^-^ emitters and higher LET-based modalities, alpha-therapy and Auger-therapy.

## Figures and Tables

**Table 1:** G0/G1, S, and G2/M populations percentages.

	G1	S	G2/M
MCF7 control	28.9	48.6	10.2
MCF7 + PIK75	32.8	40.1	11.4
MCF7 + CC115	40.3	34.2	8.6
MCF7 + doxorubicin	32.9	40.5	11.9
MCF7 + PIK75 + doxorubicin	33.6	39.1	9.65
MCF7 + CC115 + doxorubicin	33.6	37.2	11.7
MDA-MB-231 control	53.9	20.8	27
MDA-MB-231 + PIK75	52.9	21.4	27.5
MDA-MB-231 + CC115	47.8	21.7	29
MDA-MB-231 + doxorubicin	41.8	23.9	26.7
MDA-MB-231 + PIK75 + doxorubicin	50.6	19.5	27
MDA-MB-231 + CC115 + doxorubicin	45.2	29.7	24.6

**Table 2:** DNA-PK concentration.

	PIK-75	M3814	CC-115
C1	1 µM	10 µM	25 µM
C2	0.5 µM	1 µM	5 µM
C3	0.1 µM	0.1 µM	0.5 µM
C4	0.01 µM	0.01 µM	0.05 µM

## Data Availability

The original contributions presented in this study are included in the article. Further inquiries can be directed to the corresponding authors.

## Abbreviations

The following abbreviations are used in this manuscript:

DSB	Double-strand break
NHEJ	Non-homologous end joining
HR	Homologous recombination
DNA-PK	DNA-dependent protein kinase
SCLC	Small-cell lung cancer
AML	Acute myeloid leukemia
IR	Ionizing radiation
BBB	Blood–brain barrier
PSA	Prostate-specific antigen
DMSO	Dimethyl sulfoxide
PBS	Phosphate buffer solution
FBS	Fetal bovine serum
OD	Optic density
